# The influence of dapagliflozin on cardiac remodeling, myocardial function and metabolomics in type 1 diabetes mellitus rats

**DOI:** 10.1186/s13098-023-01196-6

**Published:** 2023-10-31

**Authors:** Eder Anderson Rodrigues, Camila Moreno Rosa, Dijon Henrique Salome Campos, Felipe Cesar Damatto, Gilson Masahiro Murata, Lidiane Moreira Souza, Luana Urbano Pagan, Mariana Gatto, Jessica Yumi Brosler, Hebreia Oliveira Almeida Souza, Mario Machado Martins, Luciana Machado Bastos, Suzana Erico Tanni, Katashi Okoshi, Marina Politi Okoshi

**Affiliations:** 1 Department of Internal Medicine, Botucatu Medical School, Sao Paulo State University (UNESP), Botucatu, SP Brazil; 2 LIM29, Division of Nephrology, Medical School, University of Sao Paulo, USP, Sao Paulo, SP Brazil; 3 Laboratory of Nanobiotechnology Prof. Dr. Luiz Ricardo Goulart, Institute of Biotechnology, Federal University of Uberlândia, Uberlândia, MG Brazil

**Keywords:** SGLT2 inhibitors, Streptozotocin, Papillary muscle, Ventricular function, Myocardial metabolomics, Echocardiogram

## Abstract

**Background:**

Sodium-glucose cotransporter (SGLT)2 inhibitors have displayed beneficial effects on the cardiovascular system in diabetes mellitus (DM) patients. As most clinical trials were performed in Type 2 DM, their effects in Type 1 DM have not been established.

**Objective:**

To evaluate the influence of long-term treatment with SGLT2 inhibitor dapagliflozin on cardiac remodeling, myocardial function, energy metabolism, and metabolomics in rats with Type 1 DM.

**Methods:**

Male Wistar rats were divided into groups: Control (C, n = 15); DM (n = 15); and DM treated with dapagliflozin (DM + DAPA, n = 15) for 30 weeks. DM was induced by streptozotocin. Dapagliflozin 5 mg/kg/day was added to chow. Statistical analysis: ANOVA and Tukey or Kruskal-Wallis and Dunn.

**Results:**

DM + DAPA presented lower glycemia and higher body weight than DM. Echocardiogram showed DM with left atrium dilation and left ventricular (LV) hypertrophy, dilation, and systolic and diastolic dysfunction. In LV isolated papillary muscles, DM had reduced developed tension, +dT/dt and -dT/dt in basal condition and after inotropic stimulation. All functional changes were attenuated by dapagliflozin. Hexokinase (HK), phosphofructokinase (PFK) and pyruvate kinase (PK) activity was lower in DM than C, and PFK and PK activity higher in DM + DAPA than DM. Metabolomics revealed 21 and 5 metabolites positively regulated in DM vs. C and DM + DAPA vs. DM, respectively; 6 and 3 metabolites were negatively regulated in DM vs. C and DM + DAPA vs. DM, respectively. Five metabolites that participate in cell membrane ultrastructure were higher in DM than C. Metabolites levels of N-oleoyl glutamic acid, chlorocresol and N-oleoyl-L-serine were lower and phosphatidylethanolamine and ceramide higher in DM + DAPA than DM.

**Conclusion:**

Long-term treatment with dapagliflozin attenuates cardiac remodeling, myocardial dysfunction, and contractile reserve impairment in Type 1 diabetic rats. The functional improvement is combined with restored pyruvate kinase and phosphofructokinase activity and attenuated metabolomics changes.

## Introduction

Diabetes mellitus (DM) is a growing world pandemic, leading to significant morbidity and mortality [1, 2]. Cardiomyopathy stands out from other DM complications due to the high prevalence and severity of its clinical manifestations [1]. Diabetic cardiomyopathy is defined as the presence of changes that result in cardiac remodeling with ventricular dysfunction and eventually heart failure in the absence of other conditions such as arterial hypertension, valvular heart disease, coronary artery disease and congenital heart disease [3, 4]. Heart failure may manifest as two phenotypes, preserved or reduced ejection fraction [5].

Several structural alterations have been described in clinical and experimental studies; these include cardiac hypertrophy, myocyte necrosis and apoptosis, and myocardial fibrosis. In Type 1 DM, diabetic cardiomyopathy is mainly caused by persistent hyperglycemia and reduced insulin signaling, while in Type 2 DM, cardiomyopathy is related to both hyperglycemia and insulin resistance [2, 4, 6]. The metabolic alterations induce cellular changes that result in increased inflammation and oxidative stress, calcium transient alterations, apoptosis, and extracellular collagen matrix content abnormalities [3, 4].

There is currently no specific treatment for diabetic cardiomyopathy [2]. Selective sodium-glucose cotransporter (SGLT)2 inhibitors were approved for the treatment of diabetic and heart failure patients with or without DM [7]. The primary effect of these drugs is to inhibit renal SGLT2 and decrease glucose reabsorption thus reducing plasma glucose concentration [8]. The benefits were related to a reduction in cardiovascular death or heart failure hospitalization in diabetic and nondiabetic individuals, and in heart failure patients with both preserved and reduced ejection fraction [8–12]. Cardiovascular outcomes observed in clinical trials were beyond improvement in glycemia [8, 13–15]. Despite extensive investigation, the mechanisms involved in the clinical benefits have not been completely clarified [13, 15–17]. As there is concern about SGLT2 inhibition in Type 1 DM individuals, only small studies have addressed its effects in Type 1 DM [18–21].

We have previously observed that short-term administration of SGLT2 inhibitor dapagliflozin is safe, and reduces glycemia, cardiac remodeling and oxidative stress in rats with Type 1 DM [22]. Several mechanisms have been proposed to explain the effects of SGLT2 inhibition in clinical trials. One postulated mechanism is a positive inotropic response. However, it is not known whether improved ventricular function in SGLT2-treated patients is caused by inhibition of glucose reabsorption, which induces osmotic diuresis, reducing volemia and preload and afterload [23]. This mechanism has not been properly tested [24] and experimental studies have reported both positive and negative inotropic effects after SGLT2 inhibition. In fact, empagliflozin attenuated glucose-induced increase in myocardial cytoplasmic sodium (Na^+^) and calcium (Ca^2+^) levels and enhanced mitochondrial Ca^2+^ concentration in isolated ventricular myocytes from rats and rabbits, showing that SGLT2 inhibition may normalize DM-induced changes in myocyte contractility [24, 25]. On the other hand, dapagliflozin reduced cell shortening and calcium transient amplitude in isolated ventricular myocytes from healthy and DM rats, suggesting a negative inotropic effect [26].

As SGLT2 inhibitors have been increasingly used in clinical practice [8], it is important to clarify their action in myocardial contractility. We have not identified studies analyzing the effects of SGLT2 inhibition on both myocardial and cardiac function in long-term Type 1 DM rats. Therefore, our purpose was to evaluate the effects of long-term treatment with SGLT2 inhibitor dapagliflozin on cardiac remodeling and myocardial function in rats with streptozotocin-induced DM. Our methodology using isolated papillary muscle preparations allows a direct measurement of myocardial contractility, which is independent of preload and afterload [27]. Furthermore, it permits to assess contractile reserve by subjecting papillary muscles to positive inotropic stimulation. As myocardial metabolism is changed in DM [28, 29] and gliflozins may improve metabolism [30, 31], we analyzed the activity of key enzymes involved in myocardial energy metabolism and myocardial metabolomics.

## Materials and methods

### Animals and experimental groups

Male Wistar rats were purchased from the Central Animal House at Botucatu Medical School, Sao Paulo State University (UNESP), with body weights of approximately 450 g. The study protocol (n° 1320/2019) was approved by the Ethics Committee of Botucatu Medical School, UNESP. Rats were maintained in a controlled environment (24 ± 2 ºC and 12 h light/dark cycles).

The rats were assigned to three groups:

- Control (C, n = 15): rats fed *ad libitum* with commercial chow and water;
- DM (n = 15): diabetic rats fed *ad libitum* with commercial chow and water;
- DM + dapagliflozin (DM + DAPA, n = 15): diabetic rats fed *ad libitum* with water and commercial chow supplemented with dapagliflozin.

DM was induced by intraperitoneal (i.p.) injection of streptozotocin (*Sigma Chemicals Co.*) at 40 mg/kg. Streptozotocin was diluted in 0.01 M citrate buffer pH 4.5. Control rats received the same vehicle volume i.p. Blood was collected seven days after DM induction to evaluate glycemia in a glucometer (Advantage). All rats from streptozotocin-treated groups had glycemia higher than 220 mg/dL and were included in the study.

After confirming DM status, dapagliflozin (Bristol Myers Squibb Farmacêutica S.A.) administration was initiated to the DM + DAPA group at 5 mg/kg/day added to rat chow. We had previously observed that rats with streptozotocin-induced DM at 40 mg/kg/day have a survival rate of approximately 30 weeks. Therefore, dapagliflozin treatment was maintained for 30 weeks in this long-term study. To adjust dapagliflozin dosage, chow consumption was assessed daily and body weight weekly.

### Echocardiographic evaluation

At the end of the experimental period, rats were anesthetized by an i.p. injection of ketamine (50 mg/kg) and xylazine (1 mg/kg). The exam was performed by the same researcher (KO) using an echocardiograph (General Electric Medical Systems, Vivid S6, Tirat Carmel, Israel) equipped with a 5-11.5 MHz multifrequency probe as previously described [32–34]. Structural variables were measured in a monodimensional mode from short-axis views of the left ventricle (LV) at or just below the tip of the mitral-valve leaflets and at the level of the aortic valve and left atrium. The following structural parameters were measured: LV systolic and diastolic diameters (LVSD and LVDD, respectively), LV diastolic posterior wall thickness (DPWT), and left atrium diameter (LA). LV mass (LVM) was calculated according to the formula: [(LVDD + DPWT + DSWT)^3^ − LVDD^3^] × 1.04. LV relative wall thickness (RWT) was calculated as 2 × DPWT/LVDD. LV systolic function was analyzed by ejection fraction, endocardial fractional shortening (EFS), and posterior wall shortening velocity (PWSV). LV diastolic function was assessed by early and late diastolic mitral inflow velocities (E and A waves), isovolumetric relaxation time (IVRT), and E-wave deceleration time (EDT). The myocardial performance index (Tei index) was used to jointly assess diastolic and systolic LV function. Tissue Doppler imaging (TDI) was used to evaluate mitral annulus systolic (S’) and early (E’) diastolic velocity [35–37].

### Isolated papillary muscle functional analysis

Intrinsic myocardial contractile function was assessed in isolated LV papillary muscle preparations [38, 39]. After anesthesia with pentobarbital sodium (50 mg/kg, i.p.), the rats were decapitated, and hearts removed. LV anterior or posterior papillary muscle was dissected and mounted in a chamber with Krebs–Henseleit solution at 28 ºC, oxygenated with 95% O_2_ and 5% CO_2_, pH 7.38–7.42. The composition of Krebs-Henseleit solution in mM was 118.5 NaCl; 4.69 KCl; 1.25 CaCl_2_; 1.16 MgSO_4_; 1.18 KH_2_PO_4_; 5.50 glucose and 25.88 NaHCO_3_. Force was measured by a Kyowa model 120T-20B transducer and muscle length was adjusted using a lever system. Papillary muscles were stimulated 12 times/min using electrodes that deliver 5-ms pulses at a voltage approximately 10% above threshold. After 60 min isotonic contraction, muscles were placed in isometric contraction and stretched to the apices of their length-tension curves. After 15 min of stable isometric contractions, one isometric contraction was recorded for analysis of developed tension (DT, g/mm^2^), resting tension (RT, g/mm^2^), maximum rate of tension development (+ dT/dt, g/mm^2^/s), maximum rate of tension decline (-dT/dt, g/mm^2^/s), and time to peak tension (TPT). Myocardial contractile reserve was evaluated after inotropic stimulation with 60 s post-rest contraction (PRC), increase in extracellular Ca^2+^ concentration from 1.25 mM to 2.5 mM, and the addition of β-adrenergic agonist isoproterenol (10^− 6^ M) to the nutrient solution [40, 41]. Muscle cross-sectional area was calculated by dividing muscle weight by muscle length. All force parameters were normalized for muscle cross-sectional area.

### Anatomical parameters

After removing the heart, both lungs and liver fragments were collected to assess wet-to-dry weight ratio and lung-to-body weight ratio. Wet-to-dry weight ratios were also calculated for the right ventricle and atria [42].

### Myocardial energy metabolism enzyme activities

LV samples (~ 30 mg) were homogenized in 50 mM Tris-HCl, 1 mM EDTA, protease inhibitor cocktail, and 0.1% Triton X-100, pH 7.4, using zirconium spheres (0.5 mm) for 5 min at 4 ºC in a Bullet Blender® homogenizer (Next Advance, Inc., NY, USA) [43]. The homogenate was centrifuged at 12,000 rpm for 15 min, at 4 ºC. The supernatant was used to evaluate maximum activity of key energy metabolism enzymes: hexokinase (HK, EC 2.7.1.1), phosphofructokinase (PFK, EC 2.7.1.11), pyruvate kinase (PK, EC 2.7.1.40), citrate synthase (CS, EC 4.1.3.7), and beta-hydroxyl-CoA dehydrogenase (BHADH, EC 1.1.1.35). Assays were performed in triplicate at 25 ºC; absorbance was measured every 30 s for 10 min on Synergy HT spectrophotometer (Microplate Reader, Biotek, USA). Assay buffer without sample was used as blank. Protein concentration was analyzed according to the Bradford method.

Hexokinase was assayed in a medium consisting of 75 mM Tris-HCl, 7.5 mM MgCl_2_, 0.8 mM EDTA, 1.5 mM KCl, 4 mM 2 mercaptoethanol, 0.4 mM NADP^+^, 2.5 mM ATP, 1 mM glucose, 1.4 units glucose-6-phosphate dehydrogenase, and 0.05% Triton X-100, pH 7.2. The assay was initiated by addition of glucose and monitored at 340 nm [44]. Phosphofructokinase was assayed in a buffer pH 8.2 containing of 50 mM Tris-HCl, 2 mM MgSO_4_, 5 mM KCl, 0.2 mM NADH, 1 mM ATP, 3 mM fructose-6 phosphate, 2 mM phosphoenolpyruvate (PEP), 2 U lactate dehydrogenase, 4 U pyruvate kinase, and 0.05% Triton X-100. The assay was initiated by the addition of fructose-6 phosphate and analyzed at 340 nm [45]. Pyruvate kinase was assayed in a medium with 38 mM phosphate potassium, 0.43 mM PEP, 0.2 mM ß-NAD, 6.7 mM magnesium sulphate, 1.3 mM adenosine 5’-diphosphate, 20 U lactic dehydrogenase, 1 mM fructose 1,6-diphosphate, and 0.05% Triton X-100, pH 7.4. The assay was initiated by addition of PEP and absorbance was monitored at 340 nm [46]. Citrate synthase was assayed in a medium with 50 mM Tris-HCl, 1 mM EDTA, 0.2 mM DTNB, 0.1 mM acetyl-CoA, 0.5 mM oxaloacetate, and 0.05% Triton X-100, pH 8.2. The assay was initiated by addition of oxaloacetate. Absorbance rate of change was monitored at 412 nm [47]. β-hydroxyAcyl CoA dehydrogenase was assayed in a medium with 100 mM potassium phosphate, 0.45 mM NADH, and 0.1 mM acetoacetyl CoA. The assay was initiated by addition of acetoacetyl CoA and absorbance rate of change was monitored at 340 nm.

### Metabolomic analysis

To extract metabolites, 1 mL of spectroscopic grade methanol was added to 100 mg of LV myocardium and sonicated for 15 min. Samples were macerated, homogenized, and centrifuged for 15 min at 13,000 g. The supernatant was transferred to a tube and subjected to vacuum concentrator for 60 min. The material was resuspended in 400 µL methanol and filtered through a 0.22 μm filter. Metabolite analysis was performed using an Agilent Infinity 1260/Q-TOF 6520 B coupled to an ionization source electrospray. Separation was provided by a column Agilent Poroshell 120 2.1 × 50 mm 2.7 μm. In the mobile phase, we used water acidified with formic acid (0.1% vv) (A) and methanol (B), with a gradient of 2% B , 98% B (0–6 min), and 98% B (6–12 min). Ionization parameters were nebulizer pressure of 20 psi and drying gas at 8 L/min at 220 ºC; energy of 4.5 KV was applied to the capillary. The injection volume was 4 µL.

MassHunter Qualitative software version 10.0 was used to process the raw data. A “Molecular feature extraction (MFE)” tool was used to extract the mass spectra and convert to CEF extension. Agilent Mass Profiler Professional (MPP) software version B.13.1.1 was used to filter and analyze the extracted molecule compounds. The filters used were minimum absolute abundance of 5,000 counts and all allowable charges. Analysis parameters were retention time tolerance of 0.15 min and mass window 15 ppm + 2 mDa. The molecular compounds considered were those present in 75% of samples from at least one group. Statistical analyses were performed with log2 transformed values. The unpaired T test was applied with a p-value < 0.05 and fold change ≥ 2.00. Metabolites were identified using the METLIN 2019 database.

### Statistical analysis

Normality was assessed by the Shapiro-Wilk test. Parametric variables were analyzed by one-way analysis of variance (ANOVA) followed by the Tukey test and expressed as means ± standard deviation. Non-parametric parameters were compared using the Kruskal–Wallis test followed by the Dunn test and expressed as medians and percentiles. Significance level was set at 5%.

## Results

**Fig. 1:**
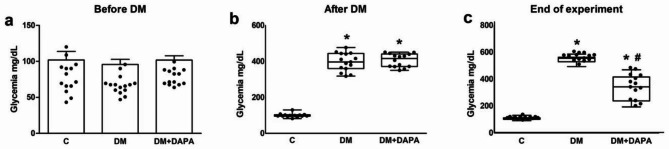
Glycemia evaluated before diabetes mellitus (DM) induction **(a)**; 7 days after DM induction **(b)**; and at the end of the experiment **(c)**. Data are expressed as individual values and means ± standard deviation or medians and percentiles. C: control (n = 14); DM: diabetes mellitus (n = 15); DM + DAPA: DM treated with dapagliflozin (n = 13); ANOVA and Tukey or Kruskal-Wallis and Dunn; * p < 0.001 vs. C; # p < 0.001 vs. DM.

One rat from C and two from DM + DAPA died during the experiment. Glycemia did not differ between groups before DM induction and was higher in DM and DM + DAPA than C after DM. All rats from DM and DM + DAPA had glycemia higher than 318 mg/dL. At the end of the experiment, glycemia was higher in DM than DM + DAPA and C (Fig. 1).

### Anatomical parameters

At the end of the experiment, body weight was lower in DM and DM + DAPA than C and higher in DM + DAPA than DM. Right ventricle weight and atria weight were lower in DM and DM + DAPA than C. The right ventricle-to-body weight ratio was lower in DM + DAPA than DM. The atria-to-body weight ratio was higher in DM than C. Lung weight was lower in DM than DM + DAPA and C. The lung-to-body weight ratio was higher in DM and DM + DAPA than C (Table 1).

**Table 1:** Anatomical variables

	C (n = 14)	DM (n = 15)	DM + DAPA (n = 13)	p value
BW (g)	574 ± 43	339 ± 31 *	413 ± 30 *^#^	< 0.001
RV weight (g)	0.29 (0.26–0.31)	0.19(0.18–0.21) *	0.21 (0.19–0.23) *	< 0.001
**RV weight/BW (mg/g)**	0.52 ± 0.07	0.57 ± 0.05	0.51 ± 0.04 ^#^	0.026
**RV weight (wet/dry)**	4.31 (4.18–4.51)	4.15 (3.97–4.23) *	4.13 (3.99–4.37)	0.023
**Atria weight (g)**	0.11 (0.10–0.12)	0.08 (0.07–0.08) *	0.08 (0.07–0.10) *	< 0.001
**Atria weight/BW (mg/g)**	0.20 ± 0.02	0.24 ± 0.02 *	0.21 ± 0.04	0.005
**Atria weight (wet/dry)**	4.79 ± 0.45	4.45 ± 0.77	4.66 ± 0.92	0.481
**Lung weight (g)**	2.33 ± 0.29	1.82 ± 0.29 *	2.11 ± 0.32 ^#^	< 0.001
**Lung weight/BW (mg/g)**	3.99 ± 0.62	5.34 ± 0.96 *	5.07 ± 0.94 *	< 0.001
**Lung weight (wet/dry)**	4.70 (4.45–4.91)	4.69 (4.43–4.81)	4.68 (4.40–5.76)	0.804
**Liver weight (wet/dry)**	3.20 (3.13–3.24)	3.21 (3.16–3.23)	3.10 (2.93–3.40)	0.614

C: control; DM: diabetes mellitus; DM + DAPA: DM treated with dapagliflozin; n: number of rats; BW: body weight; RV: right ventricle. Data are means ± standard deviation or medians and percentiles. ANOVA and Tukey or Kruskal-Wallis and Dunn; * statistically different from C; # statistically different from DM

### Echocardiographic evaluation

LV diastolic (LVDD) and systolic (LVSD) diameters did not differ between groups. LVDD-to-body weight ratio, left atrium (LA) diameter-to-body weight ratio, and LV mass index were higher in DM and DM + DAPA than C and lower in DM + DAPA than DM. LA diameter was lower in DM + DAPA than DM. LV mass was lower in DM and DM + DAPA than C. LV diastolic posterior wall thickness and relative wall thickness did not differ between groups (Table 2).

**Table 2:** Echocardiographic structural parameters

	C (n = 14)	DM (n = 15)	DM + DAPA (n = 13)	p value
**BW (g)**	574 ± 43	339 ± 31 *	415 ± 30 * ^#^	< 0.001
**LVDD (mm)**	8.64 ± 0.40	8.40 ± 0.51	8.22 ± 0.55	0.098
**LVDD/BW (mm/kg)**	14.9 (14.0–16.2)	24.2 (23.1–26.4) *	20.2 (18.6–21.9) *^#^	< 0.001
**LVSD (mm)**	4.56 ± 0.45	4.55 ± 0.54	4.60 ± 0.51	0.955
**LA (mm)**	5.87 ± 0.28	6.10 ± 0.27	5.66 ± 0.39 ^#^	0.004
**LA/BW (mm/kg)**	10.3 (9.36–10.8)	17.2 (16.4–19.8) *	13.8 (13.0–14.6) *^#^	< 0.001
**LV mass (g)**	0.89 ± 0.09	0.80 ± 0.08 *	0.76 ± 0.09 *	0.002
**LVMI (g/kg)**	1.56 ± 0.18	2.36 ± 0.32 *	1.84 ± 0.24 *^#^	< 0.001
**DPWT (mm)**	1.42 (1.39–1.46)	1.36 (1.26–1.42)	1.32 (1.28–1.36)	0.076
**RWT**	0.32 ± 0.02	0.32 ± 0.03	0.32 ± 0.02	0.681

C: control; DM: diabetes mellitus; DM + DAPA: DM treated with dapagliflozin; n: number of rats; BW: body weight; LVDD and LVSD: left ventricle (LV) diastolic and systolic diameters, respectively; LA: left atrium diameter; LVMI: LV mass index; DPWT: diastolic posterior wall thickness; RWT: relative wall thickness. Data are means ± standard deviation or medians and percentiles. ANOVA and Tukey or Kruskal-Wallis and Dunn; * statistically different from C; # statistically different from DM

LV systolic functional parameters are shown in Table 3. Posterior wall shortening velocity (PWSV) and tissue Doppler imaging for systolic velocity of the mitral annulus (average) were lower in DM and DM + DAPA than C. Tei index was higher in DM than C. Ejection fraction and endocardial fractional shortening did not differ between groups.

**Table 3:** Echocardiographic indexes of left ventricular systolic function

	C (n = 14)	DM (n = 15)	DM + DAPA (n = 13)	p value
**PWSV (mm/s)**	40 ± 3.84	34 ± 3.43 *	34 ± 3.81 *	< 0.001
**Tei index**	0.52 ± 0.05	0.58 ± 0.05 *	0.55 ± 0.04	0.004
**Ejection fraction**	0.85 ± 0.03	0.84 ± 0.04	0.83 ± 0.04	0.366
**EFS (%)**	47 ± 3.94	46 ± 5.20	45 ± 4.00	0.395
**TDI S’ (average. cm/s)**	3.56 ± 0.35	3.03 ± 0.24 *	3.15 ± 0.23 *	< 0.001

C: control; DM: diabetes mellitus; DM + DAPA: DM treated with dapagliflozin; n: number of rats; PWSV: posterior wall shortening velocity; EFS: endocardial fractional shortening; TDI S’: tissue Doppler imaging for systolic velocity of the mitral annulus (average of lateral and septal walls). Data are means ± standard deviation. ANOVA and Tukey; * statistically different from C

LV diastolic functional parameters are shown in Table 4. DM + DAPA group had lower E wave than C and lower A wave than DM and C. E/A ratio was lower in DM than C and DM + DAPA. Isovolumetric relaxation time was higher in DM than C. E wave deceleration time, tissue Doppler imaging of early (TDI E’) diastolic velocity of mitral annulus, and E wave/TDI E’ ratio did not differ between groups.

**Table 4:** Echocardiographic indexes of left ventricular diastolic function

	C (n = 14)	DM (n = 15)	DM + DAPA (n = 13)	p value
**Mitral E (cm/s)**	74 ± 5.64	69 ± 7.28	64 ± 7.00 *	0.003
**Mitral A (cm/s)**	48 ± 6.29	50 ± 9.62	39 ± 5.67 *^#^	0.002
**E/A**	1.60 ± 0.18	1.37 ± 0.27 *	1.65 ± 0.23 ^#^	0.005
**IVRT (ms)**	33 (33–37)	44 (37–44) *	37 (33–41)	< 0.001
**EDT (ms)**	52 ± 9.33	51 ± 9.21	49 ± 6.70	0.693
**TDI E’ (average. cm/s)**	3.93 ± 0.49	3.47 ± 0.56	3.52 ± 0.50	0.048
**E/TDI E’ (average)**	18.9 ± 2.53	20.1 ± 3.78	18.1 ± 3.23	0.227

C: control; DM: diabetes mellitus; DM + DAPA: DM treated with dapagliflozin; n: number of rats; E/A: ratio between early (E)-to-late (A) diastolic mitral inflow; IVRT: isovolumetric relaxation time; EDT: E wave deceleration time; TDI E’: tissue Doppler imaging of early diastolic velocity of mitral annulus (average of lateral and septal walls). Data are means ± standard deviation or medians and percentiles. ANOVA and Tukey or Kruskal-Wallis and Dunn; * statistically different from C; # statistically different from DM

### Myocardial functional evaluation

Data from functional analysis of isolated papillary muscle at basal condition are shown in Table 5. DM had lower developed tension, +dT/dt and -dT/dt than C and DM + DAPA and higher time to peak tension than C. Muscle cross sectional area did not differ between groups.

**Table 5:** Papillary muscle functional data at basal condition

	C (n = 13)	DM (n = 13)	DM + DAPA (n = 13)	p value
**DT (g/mm** ^**2**^ **)**	5.94 ± 0.83	4.82 ± 1.24 *	6.27 ± 1.12 ^#^	0.004
**RT (g/mm** ^**2**^ **)**	0.66 ± 0.09	0.69 ± 0.17	0.71 ± 0.15	0.696
**+dT/dt (g/mm** ^**2**^ **/s)**	60 ± 8.70	45 ± 11.4 *	61 ± 9.63 ^#^	< 0.001
**-dT/dt (g/mm** ^**2**^ **/s)**	28 ± 2.02	20 ± 5.10 *	25 ± 3.31 ^#^	< 0.001
**TPT (ms)**	180 (170 − 90)	210 (200–220) *	190 (185–210)	< 0.001
**CSA (mm** ^**2**^ **)**	0.95 ± 0.16	1.06 ± 0.19	0.96 ± 0.12	0.171

C: control; DM: diabetes mellitus; DM + DAPA: DM treated with dapagliflozin; DT: developed tension; RT: resting tension; +dT/dt: maximum rate of tension development; -dT/dt: maximum rate of tension decline; TPT: time to peak tension; CSA: papillary muscle cross sectional area. Data are means ± standard deviation or medians and percentiles. ANOVA and Tukey or Kruskal-Wallis and Dunn; * statistically different from C; # statistically different from DM

Table 6 shows data from isometric contraction after positive inotropic stimulation. DM had lower DT, +dT/dt and -dT/dt than C and DM + DAPA and higher TPT than C under all inotropic stimuli. DM + DAPA presented lower + dT/dt and -dT/dt than C after PRC and increase in extracellular calcium concentration.

**Table 6:** Papillary muscle functional data under positive inotropic stimulation

		C (n = 13)	DM (n = 13)	DM + DAPA (n = 13)	P value
**PRC**	**DT (g/mm** ^**2**^ **)**	9.13 ± 1.91	5.70 ± 1.70 *	7.56 ± 1.48 ^#^	< 0.001
	**RT (g/mm** ^**2**^ **)**	0.60 ± 0.17	0.68 ± 0.16	0.71 ± 0.15	0.224
	**+dT/dt (g/mm** ^**2**^ **/s)**	94 ± 23	54 ± 16 *	74 ± 12 *^#^	< 0.001
	**-dT/dt (g/mm** ^**2**^ **/s)**	37 ± 8.06	21 ± 5.31 *	26 ± 3.53 *	< 0.001
	**TPT (ms)**	187 ± 15	217 ± 18 *	204 ± 24	0.002
**2.5 mM [Ca** ^**+ 2**^ **]**_**0**_	**DT (g/mm** ^**2**^ **)**	9.03 ± 1.44	4.88 ± 2.02 *	6.71 ± 1.25 ^#^	< 0.001
	**RT (g/mm** ^**2**^ **)**	0.63 ± 0.08	0.61 ± 0.15	0.66 ± 0.13	0.666
	**+dT/dt (g/mm** ^**2**^ **/s)**	97 ± 19	50 ± 14 *	68 ± 12 *^#^	< 0.001
	**-dT/dt (g/mm** ^**2**^ **/s)**	36 ± 6.34	21 ± 5.76 *	26 ± 3.53 *^#^	< 0.001
	**TPT (ms)**	170 (165–180)	200 (190–205) *	185 (167–200)	0.003
**10**^**− 6**^ **M ISO**	**DT (g/mm** ^**2**^ **)**	7.49 ± 1.70	4.55 ± 1.24 *	6.14 ± 1.32 ^#^	< 0.001
	**RT (g/mm** ^**2**^ **)**	0.56 ± 0.15	0.56 ± 0.14	0.59 ± 0.13	0.786
	**+dT/dt (g/mm** ^**2**^ **/s)**	89 ± 23	50 ± 14 *	65 ± 25 ^#^	< 0.001
	**-dT/dt (g/mm** ^**2**^ **/s)**	48 ± 11.3	27 ± 10.6 *	36 ± 9.40 ^#^	< 0.001
	**TPT (ms)**	150 (140–150)	170 (160–185) *	160 (140–170)	0.003

C: control; DM: diabetes mellitus; DM + DAPA: DM treated with dapagliflozin; DT: developed tension; RT: resting tension; +dT/dt: maximum rate of tension development; -dT/dt: maximum rate of tension decline; TPT: time to peak tension; PRC: 60 s post-rest contraction; [Ca^+ 2^]_0_: extracellular calcium concentration; ISO: addition of isoproterenol. Data are means ± standard deviation or medians and percentiles. ANOVA and Tukey or Kruskal-Wallis and Dunn; * statistically different from C; # statistically different from DM

### Myocardial enzyme metabolism energy activities

**Fig. 2:**
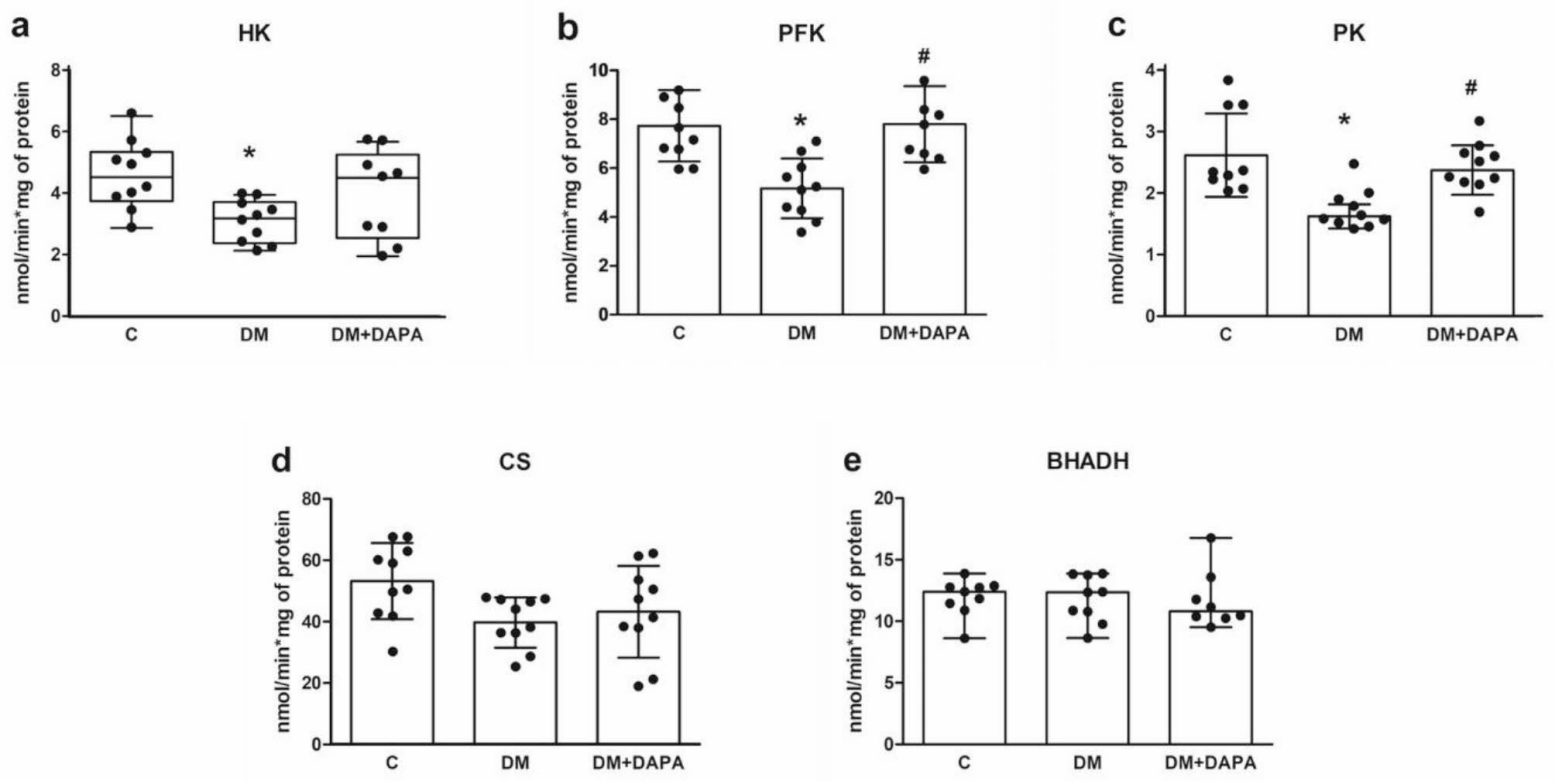
Activity of enzymes from myocardial energy metabolism. HK: hexokinase (**a**, p = 0.029); PFK: phosphofructokinase (**b**, p < 0.001); PK: pyruvate kinase (**c**, p < 0.001); CS: citrate synthase (**d**, p = 0.019); BHADH: beta-hydroxyl-CoA dehydrogenase (**e**, p = 0.364). Data are means, standard deviations, and individual values; ANOVA and Tukey

Activity of the key enzymes involved in myocardial metabolism is shown in Fig. 2. Hexokinase, phosphofructokinase, and pyruvate kinase activities were lower in DM than C. Phosphofructokinase and pyruvate kinase activities were higher in DM + DAPA than DM. Citrate synthase and beta-hydroxyl-CoA dehydrogenase did not differ between groups.

### Metabolomic analysis

**Fig. 3:**
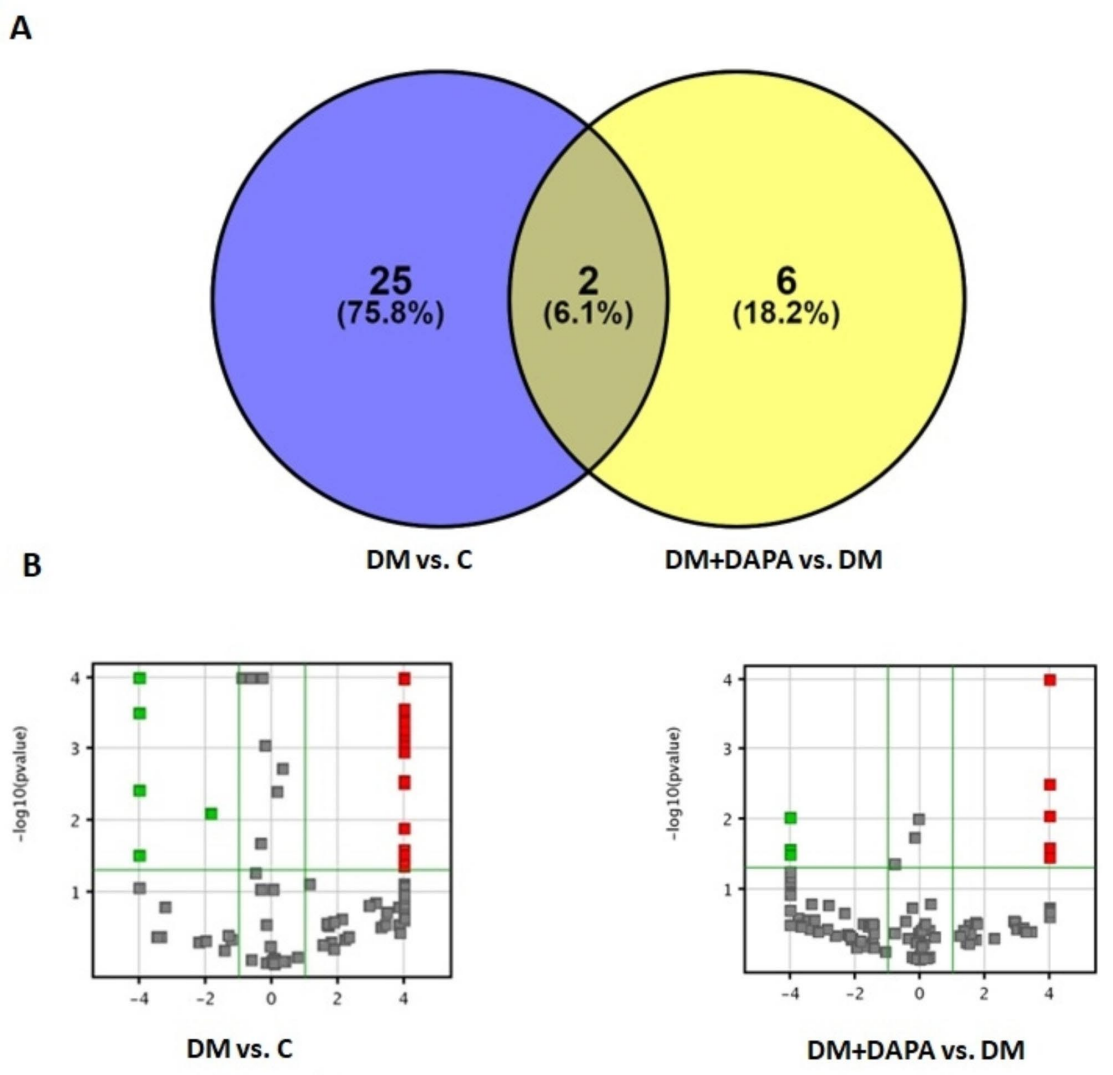
Myocardial metabolomics comparing DM vs. C and DM + DAPA vs. DM. **A:** Venn diagram; **B:** Volcano plots showing the number of differentially expressed metabolites – the most abundant shown in red and the least abundant in green

**Fig. 4:**
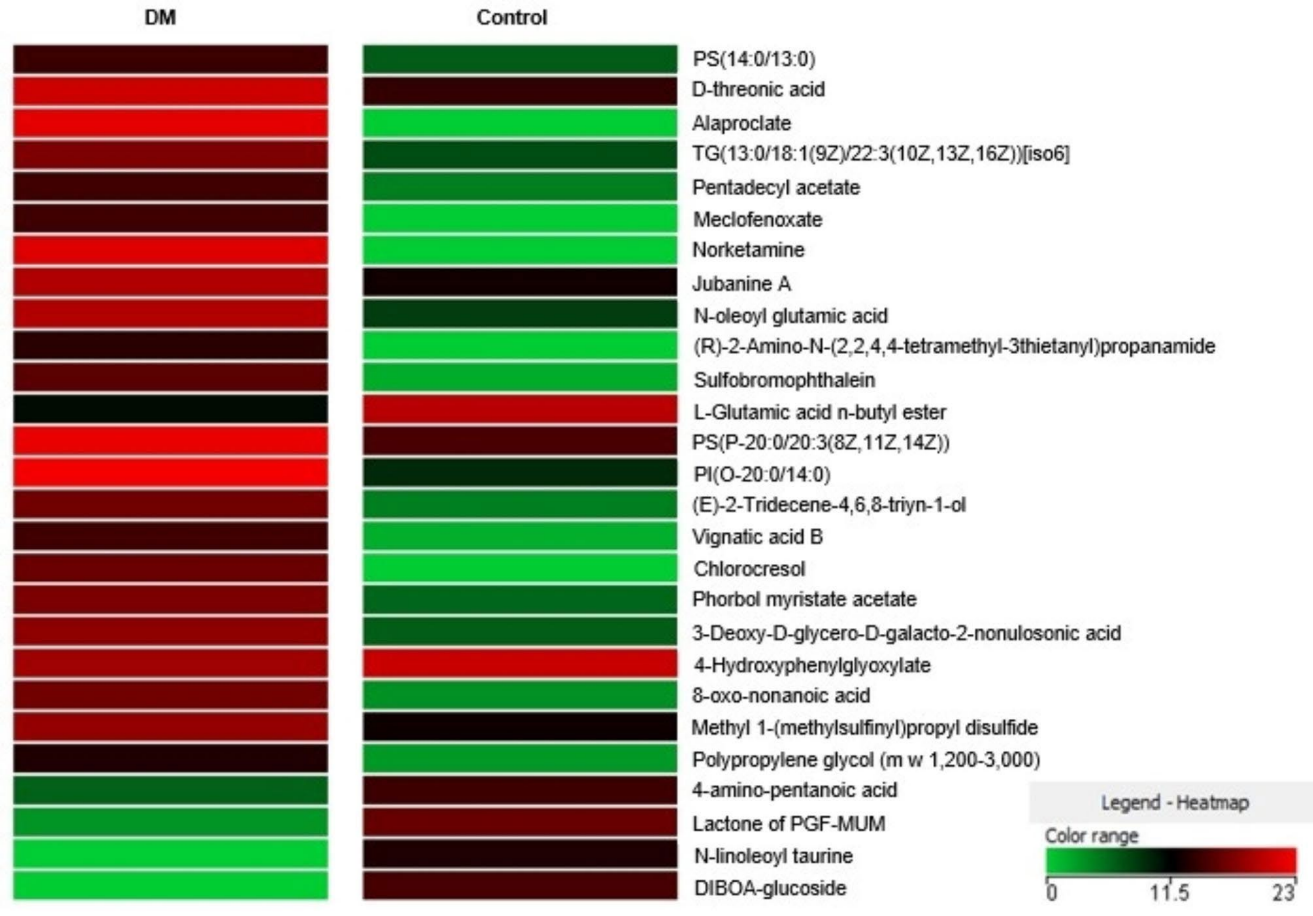
Heatmap of quantitative changes in differentially expressed metabolites between DM and C. We considered molecular compounds that were present in 75% of samples of at least one group. Fold change > 2.00: 22 compounds. Unpaired T test; p < 0.05

**Fig. 5:**
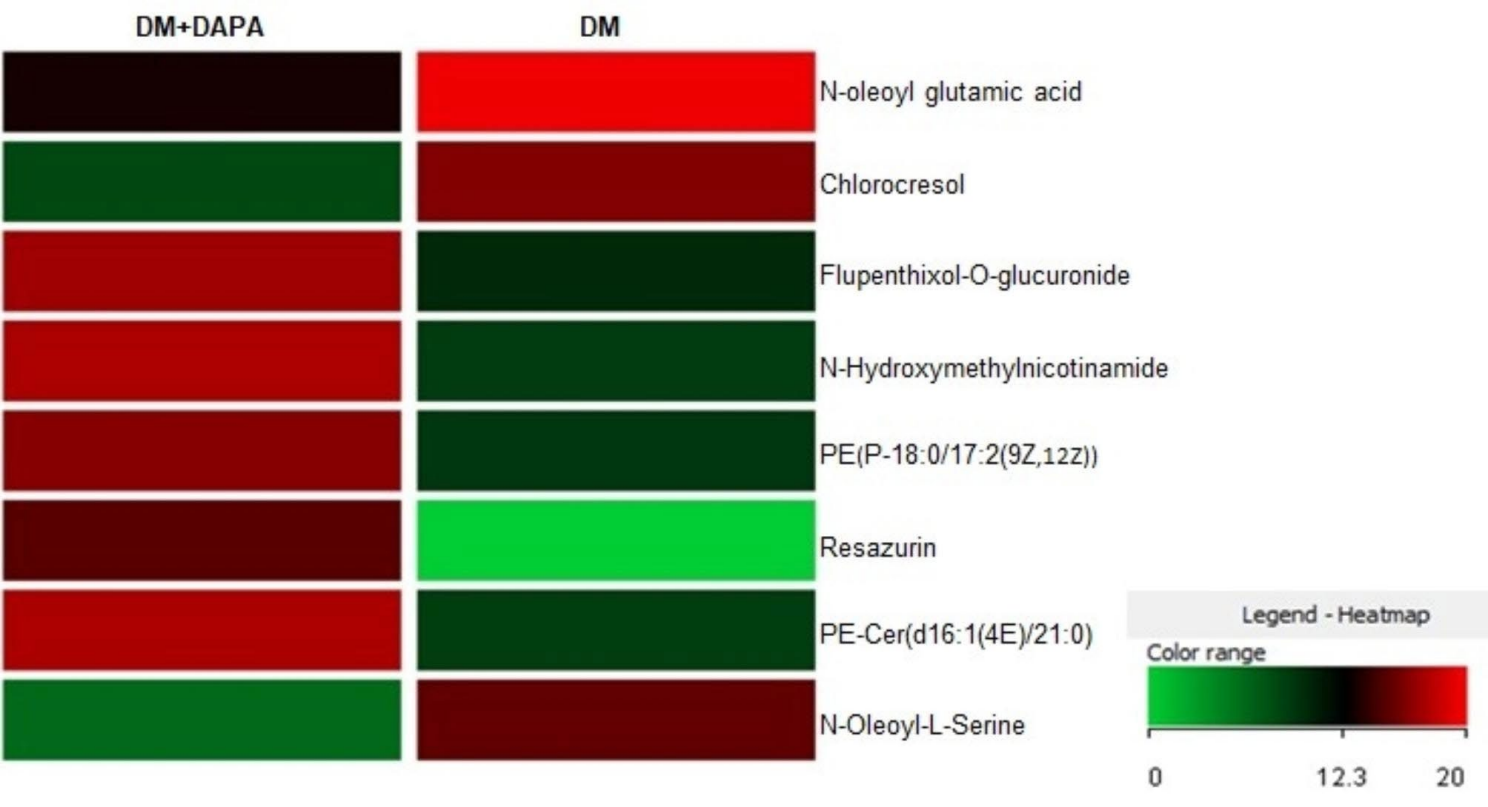
Heatmap of quantitative changes in differentially expressed metabolites between DM + DAPA and DM. We considered molecular compounds that were present in 75% of samples of at least one group. Fold change > 2.00: 8 compounds. Unpaired T test; p < 0.05

Comparisons were performed to identify differences between DM vs. C and DM + DAPA vs. DM. We identified 33 differentially expressed metabolites: 25 between DM and C, 6 between DM + DAPA and DM, and 2 differed in both comparisons (Fig. 3). The individual metabolites differentially expressed between DM vs. C and DM + DAPA vs. DM are presented in Figs. 4 and 5. Twenty-one and 5 metabolites were positively regulated in DM vs. C and DM + DAPA vs. DM comparisons, respectively; 6 and 3 metabolites were negatively regulated in DM vs. C and DM + DAPA vs. DM comparisons, respectively.

Metabolites that participated in cell membrane ultrastructure were higher in DM than C; these include *PS(14:0/13:0), TG(13:0/18:1(9Z)/22:3(10Z,13Z,16Z))[iso6]*, *PS(P-20:0/20:3(8Z,11Z,14Z), PI(O-20:0/14:0)* and *3-Deoxy-D-glycero-D-galacto-2-nonulosonic acid.* N-acylamide *N-oleoyl glutamic acid* and *chlorocresol* expressions were also higher in DM than C. Metabolites *N-oleoyl glutamic acid*, chlorocresol and *N-oleoyl-L-serine* expressions were lower and myocardial specific lipid species expressions, including phosphatidylethanolamine [PE(P-18:0/17:2(9Z,12Z))] and the sphingolipid ceramide [PE-Cer(d16:1(4E)/21:0)], were higher in DM + DAPA than DM.

## Discussion

Since the first medical agencies approval of dapagliflozin, empagliflozin, and canagliflozin for reducing glycemia in individuals with Type 2 DM, clinical indications for using SGLT2 inhibitors have substantially increased [7]. In contrast, the use of SGLT2 inhibitors in Type 1 DM is not authorized by the Food and Drug Administration [48]. Currently, SGLT2 inhibitors have been used in small clinical studies with Type 1 DM individuals [18, 19].

In this study, Type 1 DM was induced by streptozotocin. We have previously used doses between 40 and 50 mg/kg of streptozotocin to induce DM in rats [49, 50] and observed that larger doses are required in small body weight rats (data not published). As we initiated the study using rats weighing approximately 450 g, 40 mg/kg streptozotocin was sufficient to induce DM. In fact, all diabetic rats showed blood glucose higher than 318 mg/dL seven days after DM induction.

Dapagliflozin reduced blood glucose and body weight loss. This long-term result is in accordance with a previous study in which dapagliflozin was administered for 8 weeks in Type 1 DM rats [22]. Although the reduced glycemia and better cell environment may be crucial for body mass preservation, the mechanisms involved in this effect are not clear, especially considering that a major action of SGLT2 inhibitors is to decrease renal glucose reabsorption. SGLT2 was also effective in preserving skeletal muscle mass in diabetic mice [51]. To the best of our knowledge, this is the first study to use long-term dapagliflozin treatment in Type 1 DM rats.

Echocardiographic evaluation showed that DM induced left atrium dilation, and LV hypertrophy and dilation with systolic and diastolic dysfunction. These changes have often been reported in Type 1 diabetic rodents [22, 50, 52]. Dapagliflozin attenuated cardiac chamber dilation and LV hypertrophy. As a probable consequence, LV dysfunction was attenuated. Although the effects of SGLT2 inhibition on cardiac remodeling have only been evaluated in small clinical trials, data suggest it has the potential to reverse cardiac remodeling [23]. Empagliflozin reduced LV end-systolic and end-diastolic volumes in heart failure patients with reduced ejection fraction with [48] or without DM [53]. Animal models have extensively shown SGLT2 inhibition-induced improvement in cardiac remodeling [22, 54, 55]. However, the mechanisms involved in reversing pathological cardiac remodeling are not clear. It is not known whether decreased LV volume is caused by improved myocardial contractility or the diuretic effect of SGLT2 inhibitors.

We therefore analyzed myocardial function. Isolated papillary muscle preparations allow myocardial contractility evaluation without the influence of preload, afterload, cardiac chamber geometry, heart rate and extracellular environment [56]. DM reduced developed tension, +dT/dt and -dT/dt, and increased time to peak of tension in basal condition, characterizing impaired systolic and relaxation function. This result is in accordance with our previous studies on short-term DM rats [49, 57]. All these changes were attenuated by dapagliflozin. Contractile reserve was evaluated by inotropic stimulation with post-rest contraction, increase extracellular calcium concentration, and isoproterenol added to nutrient solution. The same pattern was observed after inotropic stimulation, showing that dapagliflozin attenuated myocardial contractile reserve impairment.

Several mechanisms may be involved in improved contractile function [17]. Studies have shown that SGLT2 inhibitors improve myocardial sodium and calcium homeostasis and energy metabolism, and reduces inflammation, oxidative stress, and myocardial interstitial fibrosis [17, 30]. As a probable mechanism, we analyzed the activity of key enzymes in myocardial energy metabolism and myocardial metabolomics.

Almost 60% of energy production in the healthy heart comes from fatty acid oxidation with a smaller contribution from glucose oxidation; ketone bodies and amino acids play a minor role [58, 59]. DM and cardiac remodeling exhibit contrasting metabolic changes. In DM glycolysis is impaired in DM due to lipids accumulation [60], while in cardiac remodeling the myocardial fuel changes from fatty acids to glucose [61]. Hexokinase, phosphofructokinase, and pyruvate kinase are glycolysis limiting enzymes. In this study, DM reduced the activity of these enzymes and dapagliflozin restored phosphofructokinase and pyruvate kinase activity. There are conflicting results for the effects of SGLT2 inhibitors on myocardial metabolism during cardiac remodeling. In human heart failure, empagliflozin failed to improve cardiac energetics [62]. In contrast, empagliflozin enhanced myocardial energetics in pig and rat heart failure [30, 31]. Our results suggest that changes in the activity of energy metabolism enzymes are, at least partially, involved in the dapagliflozin-induced improvement in contractility and cardiac remodeling in Type 1 DM rats.

In metabolomics, N-acylamide *N-oleoyl glutamic acid* and *chlorocresol* expression, although higher in DM than C, were lower in DM + DAPA than DM. the physiological function of N-acylamides is not completely understood; it has been suggested that they participate in metabolism control and mitochondrial respiration [63]. Phosphatidylethanolamine and ceramide were higher in DM + DAPA than DM. Changed lipid levels have been associated to development and progression of diabetic cardiomyopathy [64]. Decreased phosphatidylethanolamine content was reported in heart membranes isolated from streptozotocin-induced diabetic rats in combination with sarcolemmal Ca^2+^ transport changes [65]. In accordance with our study, dapagliflozin increased phosphatidylethanolamine concentration in renal cortex membrane of diabetic mice [66]. On the other hand, SGLT2 inhibition decreased myocardial ceramide levels in Type 2 DM rats [67]. We did not observe increased myocardial metabolite levels related to ketone bodies, an effect thought to contribute to the beneficial action of SGLT2 inhibitors. In accordance, a recent study failed to show improvement in circulating serum metabolites associated with energy metabolism in empagliflozin-treated heart failure patients [62]. We have not identified studies on cardiac metabolomics in long-term Type 1 DM rats treated with dapagliflozin.

A limitation of this study is the lack of a dapagliflozin-treated control group. However, studies have shown neutral effects in hearts and kidneys in dapagliflozin-treated healthy rats [22, 68–70]. Also, neutral effects of SGLT2 inhibition on echocardiographic parameters were well documented in healthy rats after 2, 4, 6, and 8 weeks of dapagliflozin administration [69].

## Conclusion

Long-term treatment with dapagliflozin attenuates cardiac remodeling, myocardial dysfunction, and contractile reserve impairment in Type 1 diabetic rats. The functional improvement is combined with restored phosphofructokinase and pyruvate kinase activity and attenuated metabolomics changes.

## Abbreviations

+dT/dt: Maximum rate of tension development
ANOVA: One-way analysis of variance
BHADH: Beta-hydroxyl-CoA dehydrogenase
BW: Body weight
CS: Citrate synthase
CSA: papillary muscle cross sectional area
DM: Diabetes mellitus
DM + DAPA: Diabetes mellitus treated with dapagliflozin
DPWT: Left ventricle diastolic posterior wall thickness
DT: Developed tension
dT/dt: Maximum rate of tension decline
EDT: E-wave deceleration time
EFS: Endocardial fractional shortening
HK: Hexokinase
i.p.: Intraperitoneal
IVRT: Isovolumetric relaxation time
LA: Left atrium diameter
LSVD: Left ventricle systolic diameter
LV: Left ventricle
LVDD: Left ventricle diastolic diameter
LVM: Left ventricle mass
MFE: Molecular feature extraction
MPP: Agilent Mass Profiler Professional
PFK: Phosphofructokinase
PK: Pyruvate kinase
PRC: Post-rest contraction
PWSV: Posterior wall shortening velocity
RT: Resting tension
RWT: Left ventricle relative wall thickness
SGLT2: Sodium-glucose cotransporter 2
TDI: Tissue Doppler imaging
TDI E’: Tissue Doppler imaging of early diastolic velocity of mitral annulus
TDI S’: Tissue Doppler imaging for systolic velocity of the mitral annulus
Tei: Myocardial performance index
TPT: Time to peak tension
